# Antenatal depressive symptoms among pregnant women: Evidence from a Southern Brazilian population-based cohort study

**DOI:** 10.1016/j.jad.2016.11.031

**Published:** 2017-02

**Authors:** Carolina de Vargas Nunes Coll, Mariângela Freitas da Silveira, Diego Garcia Bassani, Elena Netsi, Fernando César Wehrmeister, Fernando César Barros, Alan Stein

**Affiliations:** a Postgraduate Program in Epidemiology, Federal University of Pelotas, Brazil; b Centre for Global Child Health, The Hospital for Sick Children, Toronto, Canada; c Department of Paediatrics and Dalla Lana School of Public Health, University of Toronto, Canada; d Department of Psychiatry, University of Oxford, Oxford, UK; e Catholic University of Pelotas, Brazil

**Keywords:** Perinatal health, Depression, Pregnancy, Mental health, Epidemiology, LMICs

## Abstract

**Background:**

Antenatal depression (AD) is a major public health issue but evidence regarding its prevalence and associated factors in low and middle-income countries (LMICs) is limited. The aim of the study was to estimate the prevalence and identify risk factors for AD among Brazilian pregnant women.

**Methods:**

All women living in the urban area of the city of Pelotas, Southern Brazil, with confirmed pregnancy and estimated delivery date in the year 2015, were invited to take part. Eligible pregnant women were recruited from health services. Symptoms of antenatal depression were assessed using the Edinburgh Postnatal Depression Scale (EPDS) by face-to-face interviews. A cutoff-point of 13 or more was used to define probable AD.

**Results:**

EPDS scores were available for 4130 women. The prevalence of AD was 16% (95%CI 14·9–17·1). After adjustment for potential confounders, the factors most strongly associated with higher EPDS scores were a previous history of depression (PR 2·81; 95%CI 2·44-3·25), high parity (PR 1·72; 95%CI 1·38-2·15 - ≥2 children vs. 1 child) and maternal education (PR 5·47; 95%CI 4·22-7·09 - 0–4 vs. ≥12 years of formal education).

**Limitations:**

EPDS was administered through face-to-face interviews rather than questionnaires and some women may have felt uncomfortable reporting their symptoms leading to underreporting and consequently underestimation of the prevalence found.

**Conclusion:**

AD prevalence is substantially higher in Brazil than in high-income countries (HICs) but similar to other LMICs. Our study identified relevant risk factors that may be potential targets to plan interventions, particularly a history of depression.

## Highlights

• The proportion of women with antenatal depression (EPDS ≥13) was 16%.
• Antenatal depression is about 60% higher in Brazil than in HICs.
• Antenatal depression is mainly influenced by socioeconomic disadvantage in Brazil.
• A history of depression was the strongest predictor of antenatal depressive symptoms.
• Over 1/3 of the mothers with a history of depression reported antenatal depressive symptoms.

## 1 Background

The transition into motherhood is a challenging period that involves significant changes in the psychological, social and biological domains, and has been considered a window of increased vulnerability for the development of mental illness (Howard et al., 2014). Depression is among the most common disorders affecting women during the perinatal period (Howard et al., 2014). Most of the existing data and policies regarding perinatal mental disorders are centred on postnatal depression and less research has been carried out in relation to depression during pregnancy (Fisher et al., 2012, Howard et al., 2014).

Although antenatal depression research has been received as of lower priority, especially in low and middle-income countries (LMICs), there is evidence from high-income countries (HICs) studies that the prevalence of depressive symptoms during pregnancy is similar to or even higher than during the postpartum period. (Evans et al., 2001, Heron et al., 2004, Verreault et al., 2014) Furthermore, antenatal depression is a well-recognized predictor of postnatal depression, with almost half of episodes having their onset during pregnancy (Bennett et al., 2004, Howard et al., 2014, Robertson et al., 2004, Tachibana et al., 2015, Wisner et al., 2013). Importantly, antenatal depression has been associated with a range of negative offspring outcomes; higher risks of premature birth, low birth weight, intrauterine growth restriction, child emotional and behavioural problems, cognitive difficulties and later depression (Stein et al., 2014).

The available evidence regarding antenatal depression suggests that the point prevalence, including both major and minor depression, is around 10% in HICs (Gavin et al., 2005). Substantially higher rates have been reported in the few studies carried out in LMICs (Biratu and Haile, 2015; Faisal-Cury et al., 2009; Zeng et al., 2015). A recent systematic review on common antenatal mental disorders among women living in LMICs, reported a mean point prevalence of 15·6% (Fisher et al., 2012). However, only 8% of all LMICs had available data, and samples included in the studies were disproportionately composed of women from higher socioeconomic status and better health, limiting the generalizability of the findings.

Factors such as history of mental health problems, low maternal education, low socioeconomic status, unplanned or unwanted pregnancy, present/past pregnancy complications, intimate partner violence, recent adverse life events, lack of a partner and, lack of social support have been shown to be the main determinants of antenatal depression around the world (Howard et al., 2014; Biaggi et al., 2015) In LMICs, although research aimed at identifying women at higher risk for antenatal depression is limited compared to HICs, it is already known that women living in these settings tend to be exposed to multiple risk factors related to poverty and social adversity (Heyningen et al., 2016).

As a result of the increased risks of adverse health outcomes for both mothers and children in relation to antenatal depression, surveillance and prevention research should be a global public health priority in LMICs (Stein et al., 2014, Stewart, 2007). This is important because patterns of associations may differ from those found in HICs and accurate evidence is necessary to develop appropriate practice and policy. In Brazil, population-based estimates of antenatal depression have not been established to date. The aim of the present study is to assess the prevalence of antenatal depressive symptoms and its associated socioeconomic, demographic and health-related factors among pregnant women of a Brazilian population-based cohort study.

## 2 Methods

### 2.1 Research setting

Brazil is a large and complex country that has undergone important and rapid socioeconomic and demographic changes (economic growth, reduction in income disparities between the poorest and wealthiest populations, urbanization, improved education of women, and decreased fertility rates) in the past three decades (Victora et al., 2011). As a result of the improvement in major social determinants of health, socioeconomic inequalities to maternal-health interventions have largely decreased. (Victora et al., 2011) Despite such progress, a considerable amount of women are still living in precarious socioeconomic conditions with restricted access to health care, and many maternal-related health challenges remain. Problems such as higher rates of illegal abortions and violence against women (both emotional and physical abuse) are still very common in the country (Victora et al., 2011, Reichenheim et al., 2011).

This study was carried out in the city of Pelotas, located in the south of Brazil in the state of Rio Grande do Sul, historically one of the most affluent areas in the country. Pelotas is a medium-sized city with a current population of around 340 thousands inhabitants (93% living in the urban area), and with a gross domestic product per capita of about US$ 5390 (Instituto Brasileiro de Geografia e Estatística, 2013). As a result of European immigration (mainly Portuguese, Italian, Spanish and German), African slavery and inter-mixing of ethnic groups, the population of the city is highly diverse and admixed. However, its proportion of black people is considerably lower compared to cities located in the North and Northeast regions of the country (Instituto Brasileiro de Geografia e Estatística, 2001). In spite of being located in one of the richest states in the country, the per capita household income in the city is less than minimum wage (US$ 272) for 44.7% of the households (Instituto Brasileiro de Geografia e Estatística, 2013).

In Brazil, antenatal care is almost universal since 2006-07, reaching 98.7% of the pregnant women. Antenatal care visits generally start as soon as the woman goes to the clinic for a pregnancy test; with 83.6% of the women starting prenatal follow-up visits in the first trimester of pregnancy (Victora et al., 2011). In Pelotas, data from the 2004 Birth Cohort Study revealed less than 2% of the women without any prenatal care attendances and a mean number of visits of 8.3 during the gestational period (Barros andVictora, 2008).

### 2.2 Design and participants

This study analyzed data from the antenatal follow-up of the 2015 Pelotas (Brazil) Birth Cohort Study, a large population-based cohort study of all children born from mothers living in the urban area of the city in the year 2015. All women with confirmed pregnancy and estimated delivery dates between December/2014 and May/2016 were eligible to take part in the antenatal follow-up of the cohort. These dates were estimated taking into account two possible situations: an error range in the calculation of gestational age and preterm births. Health services that women attend for antenatal care (primary health units, university clinics, private doctors’ offices and ultrasound clinics) were contacted and visited daily (from May 2014) to identify eligible women. Different recruitment strategies were used according to the average number of women attending each of the recruitment settings monthly.

Following consent to participate, face-to-face interviews were scheduled according to the availability and needs of each participant. Trained interviewers collected data using structured questionnaires. At least one interview was carried out with the women during the pregnancy period. An initial brief interview was conducted prior to the 16th week of gestation where socioeconomic and demographic information were collected. A second and main interview was scheduled for the period between the 16th and 24th week of gestation. On this occasion, information on several maternal health pregnancy-related aspects was assessed, including antenatal depressive symptoms. For pregnant women identified after the 16th week of gestation, all data were collected at the same time.

## 3 Measures

### 3.1 Outcome variable

Antenatal depressive symptoms in the preceding week were assessed using the Edinburgh Postnatal Depression Scale (EPDS) (Cox et al., 1987). A Portuguese version of the EPDS has been previously validated in a sample of mothers from the 2004 Pelotas Birth Cohort carried out in the same setting of the present study and detailed information on the validation data can be found elsewhere (Santos et al., 2007). We used a threshold of 13 or more to define clinically significant symptoms of antenatal depression. This cutoff point has been shown to have a sensitivity of 59.6% (49.5–69.1) and a specificity of 88.3% (83.9–91.9) for depression diagnosed by clinical interviews, taken as the gold standard (Santos et al., 2007). In our study, questions were posed to the mothers by a trained interviewer, as many mothers had a low educational level and were not familiar with self-administered instruments. The administration of EPDS as an interview format is validated (Cox et al., 1987).

### 3.2 Covariates

Socioeconomic, demographic and health-related data were collected in the antenatal follow-up of the 2015 Pelotas Birth Cohort Study. Findings from systematic reviews on antenatal depression determinants were used to guide the selection of variables to be considered in our analysis. (Howard et al., 2014; Biaggi et al., 2016) The characteristics evaluated in the present study included: age (<20, 20/34, ≥35); maternal schooling (0–4/5–8/9–11, and 12 or more years of education); cohabiting with a partner (yes/no); planned pregnancy (yes/no); number of children living at home (0, 1 and 2 or more); previous history of depression (yes/no); maternal pre-pregnancy Body Mass Index (BMI) based on self-reported height and weight and categorized as underweight (<18·5 kg/m^2^), normal weight (18·5–24·9 kg/m^2^); overweight (25·0–29·9 kg/m^2^) and obese (≥30 kg/m^2^); tobacco use in the first trimester of pregnancy (yes/no); alcohol consumption in the preceding 30 days (yes/no); leisure-time physical activity in the preceding week (yes/no) and, self-reported gestational hypertension and diabetes.

In Brazil, due to historical reasons, skin color is commonly used in national surveys and epidemiological studies as a proxy of ethnic background/race, being frequently associated with social and health inequalities in the country (non-white people presenting the worse outcomes) (Melo et al., 2016, Almeida-Filho et al., 2004).

### 3.3 Data analysis

Descriptive analysis was conducted to check normality of the data and to determine the distribution of EPDS scores and the independent variables among the study sample. Because the EPDS score distribution was asymmetrical both mean and median were used to represent central tendency. Bivariate analysis was undertaken to examine the association between probable antenatal depression and potential risk factors. In continuous analysis, K tests for the comparison of medians were used, while chi-squared tests were used for the comparison of proportions. Factors found to be associated with antenatal depressive symptoms were retained in the adjusted analysis. The multivariable analysis was performed using Poisson Regression (Barros and Hirakata, 2003) according to a three-level hierarchical model determined a priori: 1) age, schooling, and skin color/race; 2) history of depression, marital status, planned pregnancy, pre-pregnancy BMI and number of children living at home; 3) smoking, alcohol consumption and physical activity during pregnancy, work outside home, hypertension and gestational diabetes. Variables that presented a p-value ≤0.20 in each level were retained in the model. Prevalence ratios (PR) and 95% confidence intervals (CI) were obtained taking into account the effect of each independent variable in relation to the outcome and adjusted for potential confounders of the same and higher levels of the model. The final model obtained was adjusted for gestational age at the time of EPDS administration. P values lower than 0.05 were considered statistically significant. Data analyses were conducted using Stata v13.1.

### 3.4 Ethical considerations

All participants gave written informed consent before the interview. For eligible participants under the age 15, written consent to participate in the study was also obtained from their parents or guardians. A list with all available mental health services in the city was provided to participants who indicated mental distress during the interviews. Psychological referral was provided only if the participant asked. The Superior School of Physical Education Research Ethics Committee from the Federal University of Pelotas approved the study under the protocol 522.064.

## 4 Results

**Fig. 1:**
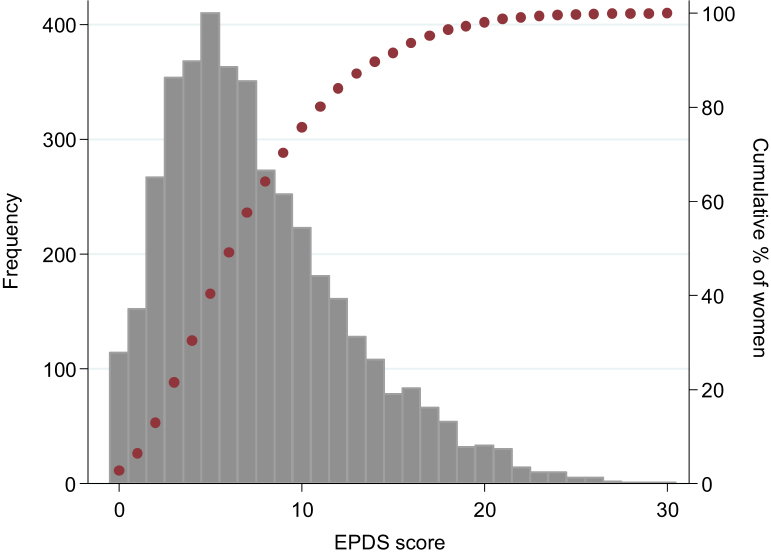
Frequency distribution of the Edinburgh Postnatal Depression Scale (EPDS) scores.

A total of 4755 eligible pregnant women were identified. Three hundred and twenty nine (6.9%) women declined to participate in the study and 12·1% of the women interviewed in the beginning of pregnancy declined to participate or were lost to follow-up at the time of the second interview. For the purpose of the present study we only included mothers with complete outcome data (n=4130). The amount of missing data was below 1·7% for most of the independent variables, except for pre-pregnancy BMI (10·4%). Participants had a mean gestation age of 23·2 weeks at the time of interview. The frequency distribution of the EPDS scores among the population is presented in Fig. 1. The mean EPDS score was 7·6 (SD 5). The median value was 7 with an interquartile range of 4–7. The distribution was asymmetrical to the right, with a skewness coefficient of 0.9 and kurtosis of 3·7; 114 (2·8%) participants scored zero in the EPDS score, and the 90th percentile was 15. The proportion of women with a positive screening for antenatal depression (EPDS ≥13) was 16% (95%CI 14·9–17·1).

Table 1 describes the proportion of women, the mean and median EPDS scores and the prevalence of probable antenatal depression according to the covariates. About 2/3 of the pregnant women were aged between 20 and 34 years, had 9 or more years of formal education and reported white skin color. Less than half of the women planned their pregnancies (46.4%) and most of them were living with a partner (83·3%). Nearly 18% of the mothers reported a previous episode of depression and about 1/5 of them were classified as obese in the pre-pregnancy period. During pregnancy, slightly less than half of the women were working outside home (47·3%) and slightly more than half were expecting their first child (53·6%). With respect to pregnancy-related behaviors, 16·9% of the women smoked during pregnancy, 12·7% reported alcohol consumption and only 16% were engaged in some leisure-time physical activity. The proportion of women who reported having gestational hypertension and gestational diabetes was 11·8% and 4·5%, respectively.

Both higher EPDS scores and antenatal depression (EPDS ≥13) were associated with: younger age, low levels of schooling, non-white skin color, higher number of children at home, not living with a partner, unplanned pregnancy, history of depression, being underweight or obese before pregnancy, smoking, alcohol consumption, no leisure-time physical activity engagement, diabetes, hypertensive and not working outside of the home (Table 1).

Prevalence ratios obtained from crude and adjusted analyses for the association between antenatal depressibe symptoms (EPDS≥13) and the covariates are presented in Table 2. Regarding the association with age, antenatal depressive symptoms were found to be associated with younger age (≤20 years) in the crude analysis, but the direction of the association changed after adjustment. Pregnant women aged ≥35 years had a 36% higher probability of experiencing antenatal depressive symptoms compared to women aged ≤20 years (PR 1·36; 95%CI: 1·06-1·73). In the adjusted model, an inverse relationship was observed between antenatal depressive symptoms and maternal schooling; women in the lowest education level (0–4 years of formal education) were about 5·5 times more likely to screen positively for antenatal depression compared to those with 12 or more years (PR =5·47; 95%CI: 4·22-7·09). For non-white pregnant women, prevalence of antenatal depressive symptoms was 28% greater than that of women in the white skin color group (PR =1·28; 95%CI 1·11–1·47).

At the second level of the hierarchical model, the probability of reporting antenatal depressive symptoms was 36% higher among those women not cohabiting with their partners (PR =1·36; 95%CI 1·16-1·60) and 34% higher among those who reported their pregnancy as unplanned (PR =1·33; 95%CI 1·14-1·57). Antenatal depressive symptoms were almost three times higher among mothers with a previous history of depression (PR 2·81; 95%CI: 2·44-3·25). A positive association between the number of children and probable antenatal depression was observed; with the prevalence of antenatal depressive symptoms being 55% higher among those who reported already having one child (PR =1·55; 95%CI 1·28-1·88) and 72% higher among women with more than two children (PR =1·72; 95%CI 1·38-2·15). Finally, at the third level of the model, the probability of presenting with antenatal depressive symptoms was about 20% and 30% higher among mothers who reported smoking (PR=1·21; 95%CI 1·04-1·42) and alcohol consumption (PR =1·30; 95%CI 1·10-1·55) during pregnancy.

## 5 Discussion

This study reported on maternal antenatal depression and associated socioeconomic, demographic and health-related factors using data from the antenatal follow-up of a large population-based cohort study in a setting of rapid socioeconomic and demographic transition in Southern Brazil. The proportion of pregnant women who screened positive for antenatal depression was 16%. This is about 60% higher than estimates reported in other studies from HICs (Bennett et al., 2004, Gavin et al., 2005). yet in accordance with the higher prevalence estimates generally reported in LMICs (Fisher et al., 2012). In Brazil, surveillance data on antenatal depression are very limited and the few existing studies were all relatively small and carried out with non-representative samples of pregnant women (Faisal-Cury et al., 2009; Fonseca-Machado Mde et al., 2015, Melo et al., 2012), limiting the comparisons with our results. In São Paulo (Southeast Brazil), a prevalence of 28·2% was reported for pregnant women but with a slightly lower cutoff point (EPDS≥12) (Fonseca-Machado Mde et al., 2015). Considering that the sample studied was comprised of low-income women in their third trimester of pregnancy, a greater prevalence than that found in our study is not surprising. A higher prevalence of depression (24·3%) was also reported for pregnant women who were attending the public health care system in the cities of Recife (Northeast Brazil) and Campinas (Southwest Brazil), using the cutoff point of 12 on the EPDS (Melo et al., 2012).

Several socioeconomic, demographic and health characteristics were found to be related to a higher risk of antenatal depression in our setting. Among the factors studied, a history of depression, low maternal education and multiparity were found to be the strongest factors associated with depressive symptoms during pregnancy. Previous depression and low maternal education are well-documented risk factors for antenatal depression and this finding is largely consistent with studies around the world (Biaggi et al., 2015, Lancaster et al., 2010). Patton et al. (2015) followed a sample of women for more than 30 years and found that 85% of those who presented with depressive symptoms during pregnancy had a history of mental health problems during adolescence or adulthood. The strong association observed in our study might reflect an increased vulnerability to depression, which may be intensified by lifestyle changes (e.g. sleeping and eating patterns) as well as physical changes (e.g. pregnancy-related symptoms and limitations) during pregnancy. Alternatively, the context of social and economic adversity experienced by a considerable amount of women living in this setting, may make them more vulnerable to depression during the perinatal period (Heyningen et al., 2016).

The prevalence of antenatal depressive symptoms in the current study was inversely related to maternal education level. Strikingly, we found that women with 0–4 years, 5–8 years and 9–11 years of formal education were 5.5, 4.3 and 2.3 times more likely to screen positively for antenatal depression, respectively, as compared to women with 12 or more years of schooling. This difference in the relative risk for depression between individuals with high versus low education within LMIC contexts has also been demonstrated in the literature (Ladin, 2008). To interpret this finding, it is also necessary to take into account that a low education level is often related to other socioeconomic disadvantages such as low income, which may be contributing to the magnitude of the association found in this study.

In the present study, women who reported a greater number of children living at home were at an increased risk of antenatal depression compared to women expecting their first child. Although some previous studies have shown similar associations, the literature is inconsistent regarding the role of parity as a risk factor for antenatal depression (Biaggi et al., 2015; Howard et al., 2014). One possible explanation for the observed associations is that childcare and the associated parenting stress experienced by those women (e.g. expectations of being able to cope with the new child) may make them more vulnerable to depression. This situation seems to be particularly relevant in the context of LMICs, where multiparity is associated with a lower socioeconomic status.

The higher prevalence of antenatal depressive symptoms observed among older mothers in this study is in line with previous research carried out in Brazil in a sample of low-income pregnant women (Faisal-Cury et al., 2009). However, the broader literature is inconsistent and also suggests higher risks of antenatal depression among adolescent mothers in HICs (Howard et al., 2014).

The association between non-white skin color and probable antenatal depression observed in this study is likely to be related to socioeconomic characteristics not assessed. In Brazil, non-white women are more likely to belong to a lower socioeconomic level, occupy poorly paid positions in the labor market, and live in areas with poorer basic infrastructure with restricted access to good quality health services (Araújo et al., 2009). Though, some studies have shown that belonging to a minority ethnic group is an independent risk factor for depression possibly due to the increased level of stress resulting from sense of discrimination (Fisher et al., 2012; Loret de Mola et al., 2016).

The higher occurrence of antenatal depressive symptoms among mothers not cohabiting with a partner and who reported an unplanned pregnancy is consistent with other research (Biaggi et al., 2015). Not cohabiting with a partner may reflect poorer social support from the partner or single parenthood, which may explain this association. In addition, an unplanned or unwanted pregnancy may place additional stress to women's lives, leading to the onset of depressive symptoms. In Brazil, an unplanned pregnancy has also been demonstrated to be an independent risk factor for persistent depression into the postpartum period (Faisal-Cury et al., 2016).

Although it is not possible to establish the directions of associations found between probable antenatal depression with smoking and alcohol consumption during pregnancy due to the cross-sectional nature of the data, our findings are in line with the literature showing that mothers with mental health problems are more likely to use alcohol and tobacco (Leis et al., 2012; Smedberg et al., 2015; Faisal-Cury et al., 2009). On the other hand, despite recent evidence showing that leisure-time physical activity practice during pregnancy has the potential to decrease maternal depressive symptoms, we did not find this association in the presente study (Shivakamur et al., 2011).

### 5.1 Strengths and limitations

Although the EPDS is a widely used and accepted screening instrument to evaluate the presence of perinatal depressive symptoms, the lack of a confirmed clinical diagnosis of antenatal depression can be considered a limitation of this study. Furthermore, the EPDS was administered through face-to-face interviews rather than questionnaires and some women may have felt uncomfortable reporting their symptoms. This may have led to underreporting and consequently underestimation of the prevalence found. Another limitation is that antenatal depressive symptoms were assessed at different time points for women identified after the standard period of data collection (16–24 weeks) and this may have influenced the prevalence found. However, while examining the association between antenatal depression and related factors we adjusted our analysis for gestational age to overcome this limitation. It should also be noted that, in spite of near universal coverage in antenatal care among women living in the setting studied (Victora et al., 2010), it is possible that a very small percentage of women who did not attend antenatal care until the end of pregnancy were not included in the study sample. Therefore, because losses are more likely to have occurred among pregnant women from a low socioeconomic status (Victora et al., 2010), a subtle underestimation in the prevalence of antenatal depression observed in our study would be expected. Lastly, the lack of assessment of some known risk factors for mental distress in the context of LMICs (e.g. intimate partner violence, adverse life events and household wealth), can be considered a limitation of the present study as they could add important evidence to inform the development of prevention and health promotion strategies.

Despite the limitations, as far as we are aware, this is the first large population-based study to report on the prevalence of antenatal depression and its related factors among pregnant women living in a middle-income country. The very low refusal rate and the high follow-up rate provide confidence in the statistical estimates reported in the study.

## 6 Conclusions

A relatively high prevalence of antenatal depressive symptoms was found among Southern Brazilian women. Considering that Brazil is a large country with high rates of socioeconomic inequalities it is likely that the prevalence of depression among pregnant women living in the poorest areas of the country may be even higher. Overall, the data indicated that pregnant women living in this setting are exposed to multiple risk factors to antenatal depression. Factors related to socioeconomic disadvantage together with previous history of depression were the main predictors of antenatal depressive symptoms among this population. Intervention strategies focused on identification, prevention and management of antenatal depression should be urgently implemented in the standard routine prenatal care system in Brazil since perinatal mental health services are still very limited. Given that over 1/3 of the mothers with a previous history of depression will face clinically significant antenatal depressive symptoms, policies aimed at identifying pregnant women with a history of depression are critical to early intervention and may mitigate the potentially negative consequences of antenatal depression for maternal-child health.

## Role of funding source

This study is based on data from The 2015 Pelotas (Brazil) Birth Cohort Study which is currently supported by the Wellcome Trust (grant 095582/z/11/z), the Brazilian National Research Council (CNPq) and the Coordination for the Improvement of Higher Education Personnel (CAPES).

## Figures and Tables

**Table 1:** Characteristics of the population according to socioeconomic, demographic and health-related variables, and its association with overall EPDS scores and probable antenatal depression (EPDS ≥13). Pelotas, Brazil, 2015.

**Variables**	**N (%)**	**Mean EPDS (CI 95%)**	**Median (25–75)**	**P**^a^	**Antenatal depression N (%)**	**p**
**Mother´s age (years)**				<0.001		0.01^b^
<20	603 (14·6)	8·6 (3.6–13.6)	8 (5–11)		121 (21·0)	
20–34	2950 (71·4)	7·5 (2.6–12.4)	6 (4–10)		450 (15·3)	
≥ 35	577 (14·0)	7·3 (2.1–12.5)	6 (4–10)		90 (15·6)	
**Schooling (years)**				<0.001		<0.001^c^
0–4	353 (8·6)	10·5 (4.8–16.2)	10 (6–14)		124 (35·1)	
5–8	941 (22·8)	9·3 (3.9–14.7)	9 (5–13)		247 (26·3)	
9–11	1445 (35·0)	7·5 (2.8–12.2)	7 (4–10)		204 (14·0)	
≥12	1389 (33·7)	5·8 (1.8–9.8)	5 (3–8)		86 (6·2)	
**Skin color**				<0.001		<0.001^b^
White	2901 (70·3)	7·1 (2·3–11·9)	6 (4–10)		385 (13·3)	
Non-white	1224 (29·7)	8·8 (3·6–14.0)	8 (5–12)		276 (22·6)	
**Living with partner**				<0.001		<0.001^b^
Yes	3442 (83·3)	7·3 (2·5–12·1)	6 (4–10)		496 (14·4)	
No	688 (16·7)	9·0 (3·4–14·6)	8 (5–12)		165 (24·0)	
**Planned pregnancy**				<0.001		<0.001^b^
Yes	1916 (46·4)	6·7 (2·1–11·3)	6 (3–9)		216 (11·3)	
No	2212 (53·6)	8·3 (3·1–13·5)	7 (4–11)		444 (20·1)	
**Smoking**				<0.001		<0.001^b^
Yes	696 (16·9)	10·0 (4·6–15·4)	9 (6–13)		119 (29·5)	
No	3434 (83·2)	7·6 (2·7–12·5)	7 (4–10)		258 (16·1)	
**Alcohol consumption**				<0.001		<0.001^b^
Yes	525 (12·7)	8·9 (3·5–14·3)	8 (5–12)		125 (23·8)	
No	3604 (87·3)	7·4 (2·5–12·3)	6 (4–10)		536 (14·9)	
**Work outside home**				<0.001		<0.001^b^
Yes	1953 (47·3)	6·8 (2·3–11·3)	6 (4–9)		219 (11·2)	
No	2176 (52·7)	8·3 (3·0–13·6)	7 (4–11)		442 (20·3)	
**Number of children**				<0.001		<0.001^c^
0	2213 (53·6)	6·9 (2·4–11·4)	6 (4–9)		250 (11·3)	
1	1329 (32·2)	7·9 (2·8–13·0)	7 (4–11)		241 (18·1)	
2 or more	588 (14·2)	9·6 (3·6–15·6)	9 (5–13)		170 (28·9)	
**Hypertension**				<0.001		<0.001^b^
Yes	485 (11·8)	8·9 (3·7–14·1)	8 (5–12)		114 (23·5)	
No	3621 (88·2)	7·4 (2·5–12·3)	6 (4–10)		542 (15·0)	
**Diabetes**				<0.001		0.002^b^
Yes	181 (4·5)	9·0 (3·4–14·6)	8 (5–12)		43 (23·8)	
No	3811 (95·5)	7·5 (2·6–12·4)	6 (4–10)		604 (15·6)	
**History of depression**				<0.001		<0.001^b^
Yes	737 (17·9)	10·5 (4·6–16·4)	10 (6–14)		257 (34·9)	
No	3389 (82·1)	7·0 (3·5–11·5)	6 (4–9)		404 (11·9)	
**Physical activity**				<0.001		
Yes	670 (16·5)	6·4 (1·8–11·0)	6 (3–9)		65 (9·7)	<0.001^b^
No	3396 (83·5)	7·8 (2·8–12·8)	7 (4–11)		585 (17·2)	
**Pre-pregnancy BMI**				0.013		0.044^b^
<18.5	117 (3·2)	7·8 (2.6–13.0)	6 (4–11)		21 (18·0)	
18·5–24·9	1820 (49·2)	7·3 (2.5–12.1)	6 (4–10)		259 (14·2)	
25·0–29·9	1035 (27·9)	7·3 (2.5–12.1)	6 (4–10)		149 (14·4)	
≥ 30	727 (19·7)	8·0 (2.9–13.1)	7 (4–11)		133 (16·3)	

a K test for the comparison of medians.

b Chi-squared test for heterogeneity.

c Chi-squared test for linear trend.

**Table 2:** Crude and adjusted analysis of the association between antenatal depressibe symptoms (EPDS≥13) and socioeconomic, demographic and health-related characteristics. Pelotas, Brazil, 2015.

**Variables**	**Crude PR (95% CI)**	**P**^b^	**Adjusted PR (95% CI)**^a^	**P**^b^
***Level 1***				
**Mother´s age (years)**		0.01		0.037
<20	1		1	
20–34	0.76 (0·63–0·91)		1.21 (1.01–1.45)	
≥35	0.78 (0·61–1·00)		1.36 (1.06–1.73)	
**Schooling (years)**		<0.001		<0.001
0–4	5·67 (4·42–7·28)		5·47 (4·22–7·09)	
5–8	4·24 (3·36–5·34)		4·25 (3·34–5·42)	
9–11	2·28 (1·79–2·90)		2·27 (1·78–2·90)	
≥12	1		1	
**Skin color**		<0.001		0.001
White	1		1	
Non-white	1·70 (1·48–1·95)		1·28 (1·11–1·47)	

***Level 2***				
**Living with partner**		<0.001		<0.001
Yes	1		1	
No	1·66 (1·42–1·95)		1·36 (1·15–1·61)	
**Planned pregnancy**		<0.001		0.001
Yes	1		1	
No	1·78 (1·53–2·07)		1·33 (1·13–1·57)	
**Number of children at home**		<0.001		<0.001
0	1		1	
1	1·61 (1.36–1.89)		1·55 (1·28–1·88)	
2 or more	2·56 (2.15–3.00)		1·72 (1·38–2·15)	
**History of depression**		<0.001		<0.001
Yes	2.93 (2.56–3.35)		2.81 (2.44–3.25)	
No	1		1	
**Pre-pregnancy BMI**		0.044		0.444
<18.5	1		1	
18.5–24.9	0.79 (0.53–1.19)		0.90 (0·63–1·29)	
25.0–29.9	0.80 (0.53–1.21)		0.87 (0·60–1·27)	
≥ 30	1·02 (0·67–1·55)		1·00 (0·69–1·46)	

***Level 3***				
**Smoking during pregnancy**		<0.001		0.012
Yes	2·17 (1·88–2·51)		1·22 (1·05–1·43)	
No	1		1	
**Alcohol during pregnancy**		<0.001		0.002
Yes	1·60 (1·35–1·90)		1·30 (1·10–1·55)	
No	1		1	
**Work outside home**		<0.001		0.540
Yes	1		1	
No	1·81 (1·56–2·10)		0.95 (0·82–1·11)	
**Hypertension**		<0.001		0.123
Yes	1·57 (1·31–1·88)		1·15 (0·96–1·38)	
No	1		1	
**Diabetes**		0.002		0.184
Yes	1·53 (1·16–2·00)		1·19 (0·92–1·55)	
No	1		1	
**Leisure-time physical activity**		<0.001		0.170
Yes	1		1	
No	1·78 (0·44–0·78)		1·18 (0·93–1·49)	

a PRs shown for the adjusted analysis are adjusted for variables presenting a P value <0.20 in the same or in the upper levels of the conceptual model.

b Wald test for heterogeneity.
